# The Role of Family in a Dietary Risk Reduction Intervention for Cardiovascular Disease

**DOI:** 10.3390/healthcare4040074

**Published:** 2016-09-30

**Authors:** Tracy L. Schumacher, Tracy L. Burrows, Deborah I. Thompson, Robin Callister, Neil J. Spratt, Clare E. Collins

**Affiliations:** 1 School of Health Sciences, Faculty of Health and Medicine, University of Newcastle, Callaghan 2308, NSW, Australia; tracy.burrows@newcastle.edu.au (T.B.); clare.collins@newcastle.edu.au (C.C.); 2 Priority Research Centre for Physical Activity and Nutrition, University of Newcastle, Callaghan 2308, NSW, Australia; robin.callister@newcastle.edu.au; 3 USDA/ARS Children’s Nutrition Research Centre, Baylor College of Medicine, Houston, TX 77030, USA; dit@bcm.edu; 4 School of Biomedical Sciences and Pharmacy, University of Newcastle, Callaghan 2308, NSW, Australia; 5 Priority Research Centre for Translational Neuroscience and Mental Health & Faculty of Health and Medicine, University of Newcastle, Callaghan 2308, NSW, Australia; neil.spratt@newcastle.edu.au; 6 Hunter New England Local Health District & Hunter Medical Research Institute, New Lambton Heights 2305, NSW, Australia

**Keywords:** cardiovascular, family, health behaviour, nutrition, protection motivation theory

## Abstract

Diet is an essential strategy for the prevention of primary and secondary cardiovascular disease (CVD) events. The objectives were to examine: how families at increased risk of CVD perceived personal risk, their motivations to make dietary changes, their understanding of diet, and the influence of other family members. Individuals (>18 years) who completed an Australian family-based CVD risk reduction program were invited to a semi-structured telephone interview. Responses were recorded, transcribed verbatim and analysed using a systematic deductive approach with coding derived from key concepts developed as part of the interview structure. Seventeen participants from eight families were interviewed (aged 18–70 years, 47% male, five with CVD diagnosis). Key themes indicated both intrinsic and extrinsic motivations to improve heart health, variations in risk perception, recognition of the role diet plays in heart health, and the extent of family influences on eating patterns. Discrepancies between perceived and actual CVD risk perception impacted on perceived “need” to modify current dietary patterns towards heart health recommendations. Therefore, strategies not reliant on risk perception are needed to engage those with low risk perception. This could involve identifying and accessing the family “ringleader” to influence involvement and capitalising on personal accountability to other family members.

## 1. Introduction

Cardiovascular disease (CVD) is a leading cause of morbidity and mortality in Australia and worldwide [1,2]. Modifiable behaviours related to risk factors include diet, exercise, smoking, and alcohol consumption [3]. Diet is effective in reducing CVD event rates, but altering eating patterns can be challenging [4,5,6]. Many believe their cardiovascular disease is due to bad luck or heredity, or is a short-term problem [7,8]. Factors such as social support and unhelpful social contacts can affect the motivations of those with CVD, and contribute to poor adherence to dietary recommendations [9,10,11]. Family interactions are also an important influence on adherence to chronic disease recommendations and may offer practical, emotional and social support, or be at a similar stage of change for improving health behaviours [10,12,13,14]. Alternatively, family interactions can act as a negative pressure and function as barriers to improvements [10,12].

The inclusion of family members in dietary counselling for CVD risk reduction was trialled in the ‘Love Your Food, Love Your Heart, Love Your Family’ (FHF) pilot randomised controlled trial [15]. This intervention was based on Protection Motivation Theory (PMT), as it has been shown to be applicable to CVD prevention [16]. PMT proposes that people act in accordance with their estimation of threat and coping appraisal [17]. This theory parallels other integrated theories, such as the Integrated Theory of Health Behavior Change [18]. Threat appraisal corresponds with knowledge and beliefs, while coping appraisal shares similarities with components of self-regulation, beliefs and social facilitation. Together, these are posited to lead people to act in accordance with the threat estimation or to engagement in self-management behaviours [17,18]. For this intervention, it was hypothesized that families with a moderate to high CVD risk would perceive their threat of adverse CVD events as high and be highly motivated to reduce their risk. Families were eligible to participate in the FHF study if at least one 18–70 year old family member (index person) had experienced an adverse CVD event or was classed at or above moderate risk using Australian cardiovascular risk charts [19]. Families were required to attend two sessions (one baseline; one at 12 weeks) together as a group that included assessments and feedback, with counselling provided in the first session only. Blood lipid profiles, anthropometric measures and current dietary intakes were assessed on all participating family members; these data were used to provide feedback in a single 45-min, family-based, dietetic counselling session with an Accredited Practising Dietitian. The dietary advice (1) recommended increased consumption of foods from the Mediterranean and Portfolio diets readily available in the Australian food supply (2) and reduced consumption of less healthy foods [4,20]. Control families were waitlisted for three months and then were invited to undergo the intervention. Recruitment proved challenging; a total of 23 families were recruited over an 18-month period from a variety of sources, ranging from flyers displayed in the university setting, to media releases, accessing a volunteer register and attending cardiac rehabilitation classes and stroke clinics. Dietary changes made during the intervention aligned with the eating patterns recommended and were beneficial, though small or incremental in magnitude. These included reductions in usage of whole-fat milks, cheese and meat products.

Follow-up interviews were conducted to gain qualitative insights from the index participants and their family members to inform future family-based studies. For example, gaining an understanding of the participants’ risk perceptions and motivations to join a dietary CVD prevention study could be utilized to improve on future recruiting methods and assist with long-term behaviour change. Appreciation of and preparation for individual influences on family eating patterns may inform strategies that clinicians undertake to support individuals and their families towards adopting dietary recommendations.

Therefore, the aim of the current study was to examine the index participants and their family members who agreed to participate in the interviews (1); motivations for joining a dietary family-based study for CVD prevention; (2) perceptions of CVD risk in self and other family members; and (3) understanding of the role of diet and experiences of individual or family-based influences on eating patterns.

## 2. Materials and Methods 

This study is a qualitative exploration of the FHF study, as more understanding was required in relation to risk perception and the role of family in eating patterns related to CVD health [15]. This study was conducted in the Hunter region of New South Wales, (Australia), with a population of approximately 160,000 people and median weekly income of $AUD 1165 (2011 data). A qualitative design was selected to elicit rich information and insights from participant experiences. Adults who completed the intervention phase of the pilot FHF study were invited by mail to participate in individual semi-structured telephone interviews following collection of final data. Detailed methods for recruitment for the initial pilot phase have been described previously [15]. The need for interviews was explained to the participants as “…the study raised other questions about barriers and family dynamics that investigators feel needs further investigating”. Interviews were performed by one researcher (Tracy L. Schumacher) post intervention at the convenience of the participants.

Telephone interviews were chosen as the preferred method of data collection, as, although there is an acknowledged loss of non-verbal data, a relationship was already established between the researcher and individual interviewees [21]. In addition, as participants were free to choose their own interview time and location for the telephone call, privacy from other family members was optimized, and, therefore, they were more willing to disclose sensitive information [21]. An interview guide was developed primarily by two authors (Tracy L. Schumacher and Deborah I. Thompson) that included a prepared script to introduce the study; questions were based on issues that emerged from observation during the pilot study (see Supplementary materials) [21]. Probes and prompts were used to expand and clarify responses, and notes were taken by the interviewer throughout to ensure all questions were answered [22]. The areas of inquiry included motivations for participating in the FHF study, concerns about heart health, perceptions regarding the role of diet in heart health, barriers to healthier eating, and how family dynamics influenced dietary decisions. Interviews on a topic were continued until no new information emerged.

The interviews were digitally recorded with the participants’ consent and transcribed verbatim. Qualitative analyses were conducted by an independent experienced qualitative researcher using NVIVO 10 and expanded on by the lead author (Tracy L. Schumacher) [23]. A systematic deductive approach to data analysis was adopted. Initial codes formulated from the overall study aim to develop a taxonomy of the domains that characterised the multifaceted experiences of participants and relationships within the data. This was revised and further expanded after coding of additional transcripts. Following coding of all the transcripts, codes were examined and collapsed into categories by an external qualitative researcher (Vibeke Hanson). Themes were identified and summarised by the lead author (Tracy L. Schumacher) in consultation with the research team (Tracy L. Burrows, Deborah I. Thompson, Robin Callister, Neil J. Spratt and Clare E. Collins). Ethics approval was obtained from the University of Newcastle Human Research Committee, H-2012-0246.

## 3. Results

Seventeen program participants from eight families were interviewed a mean of 1.5 months post intervention (acceptance rate of 59%). Participants were invited to participate in interviews as they completed their interventions until no new information emerged. Participants were aged 18 to 70 years (mean ± SD, 53 ± 6 years), 47% men, with two to five participating members per family. Five interviewees were index participants, two of which had previously experienced adverse CVD events. The remaining three index participants had undertaken preventive surgery. The median reported income category of the index interviewees was $AUD 500–699 per week (range from no income to $AUD 2500–2999), lower than the regional median. This was also lower than the reported median income ($AUD 700–999, range $AUD 300–499 to $3000+) of other FHF index participants who were not involved in the qualitative interviews. The remainder of the interviewees were family members (five spouses, five children, one sibling and one grandchild); and one family member also had a diagnosed CVD condition. All spouses lived with the index person. Only two of the children interviewed resided with their parents, with the remaining three, and the grandchild, in their own established residence. Interviews lasted approximately 21 min(range 16–29 min). Key findings were: the family unit was important for motivation; risk perceptions were varied and not necessarily based on objective measures; diet was seen as important, albeit in the context of other lifestyle factors and; family strongly influenced individual dietary eating patterns. A summary of salient points that emerged from the areas of enquiry can be seen in Table 1.

### 3.1. Motivations

Motivations to join the study encompassed two broad areas: the importance of the study being inclusive of the family unit and involvement due to a more dominant family member.

Involvement of the family unit as a motivator for dietary and lifestyle changes was identified to have important benefits through demonstrating supportiveness or as mutually-implied inter-family accountability. It was alluded to that dietary changes for a single individual would be challenging and the role of support either given or received would be of value. As several participants said:

“*There’s not much point in one person being healthy in the family when there is heart disease in that side of the—the husband’s side of the family. So it needed to be like a joint—a whole family thing.*”

(female)

“*Because there were so many members in the family that had had heart issues, I felt that we would be a good source of information and we might be able to help each other.*”

(male)

“*So any changes in your lifestyle, like I’m not good at that, but if I’m accountable to other people, I’m better at it.*”

(female)

All interviewed families clearly identified 161–162 a family "ringleader" who was instrumental in getting their family to participate in the Love your food, Love your heart, Love your family intervention. “*… after having had a heart attack himself, he has been the driving force*”. Ringleaders were not from any particular family position: two fathers, one husband, one wife, two daughters and one grandfather were identified as performing this role within the family. The grandfather, fathers and husbands were all index participants, with the wife and daughters being secondary recruits. “Ringleaders” varied in their motivations for participating, with reasons ranging from a long-term interest in improving their diet to a desire to bring about positive changes in health for themselves and their family.

“*Oh my God, I was just so excited, I was so excited... this was just a great opportunity to…include some family members because some…weren’t eating very well at all…Look my husband probably resisted the most... My mother was very excited. My daughter was okay; she just wanted to join in. My husband doesn’t like change … he thought if it meant having to eat things he didn’t like then he would never manage, but we talked him into it.*”

(female)

While many of the “secondary recruits” were motivated to participate to support their family or help improve the health of a relative with heart health issues, they also enrolled for personal reasons, such as to reduce their personal risk of heart disease or other lifestyle diseases. In some instances, these ‘secondary recruits’ appeared to have a higher level of readiness to adopt dietary and lifestyle changes.

“*Well, I just thought something that would improve my diet and assess my health was important, because I knew my diet wasn’t very good*”.

(female)

“*…seeing both my parents and my in-laws, just the amount of medication that they take.*”

(female)

### 3.2. Risk Perceptions

Risk perceptions, as shown through concern levels about heart health for the index participants who had experienced adverse CVD events, varied substantially. One participant was very concerned about his heart health:

“*Well it’s close to maximum because I have had previous heart surgery…My father died with heart trouble so I’d want to be very conscious about the heart.*”

(male)

In contrast, others expressed no real concern for heart health, despite previous surgery or CVD events, and viewed their heart health as of no more concern than those without a similar medical history. This appeared to be the result of confidence in their medical and surgical treatment, with little need for ongoing preventive strategies.

“*[I’m] not concerned, I’ve sort of had my operations and all my medication, I’m quite happy with the way it appears to be going at the moment.*”

(male)

Some diminished the severity of their condition due to their treatments, as suggested by the use of ‘moderating words’.

“*Well, it was a slight heart attack, they put a stent in which wasn’t a great big operation and I am on medication now, that was three years ago, three and a half years ago and it has not annoyed me, in any way.*”

(male)

Another used moderating words to describe adverse CVD events of close family members and indicated he only recognised his individual risk following his own event:

*Q: “Were you aware of your risk?*”

*A: “…I was aware through hearing of different family members having their little heart attacks or bypasses or dying from it, but did that register with me? No. ….As soon as it happened [his own adverse CVD event], then it was a reminder that “Oh well, you lost a cousin when he was 32 and don’t forget your brother…had a heart attack, or had a bypass and your Dad did, and then your Aunty”, and it’s like “oh yeah, that’s right. No wonder we’re at risk.*”

(male)

Thus, overall, for some participants, there was not a strong or consistent association between their perception of CVD risk and objective evaluation of risk based on assessments and medical history.

For the family members who also joined the study as secondary recruits, CVD risk perception was influenced by self-justification of lifestyle and heredity factors, although a healthy diet was generally acknowledged to play a role in decreasing CVD risk by all interviewees. Some family members rationalised their level of concern by factors such as the presence of symptoms or family history of heart-related problems:

“*…No. I’m not very concerned about my heart. I seem to have kind of lowish to medium blood pressure and I don’t seem to have any problems as yet. Nothing’s come up on any tests or anything like that.*”

(female)

“*…they’ve got some of the genes of their father, who’s got the heart disease in the family. Maybe because I haven’t, I’m just, you know, a bit more – not as concerned about me.*”

(female)

For four other supportive family members, justification for reduced heart health concern was given as adherence to healthy dietary habits in combination with other preventive behaviours:

“*I know that if I’m to remain healthy I’ve got to not only eat the right stuff, but I’ve got to keep moving.*”

(male)

“*Because I know I do eat well and I know I’m on medication and my husband’s on medication, but I thought we had a reasonable diet. So I wasn’t that concerned about heart problems.*”

(female)

### 3.3. Heart Health and Diet

All participants rated the relationship between diet and heart health as moderate to high. All conveyed an awareness of the contribution of diet to heart health. The colloquial “*you are what you eat*” was a common analogy used to express this strongly-perceived association. Others believed in the importance of moderation and balance as the crucial element of a healthy diet: “*too much of a good thing is not good for you*”.

Many specifically expressed an awareness of factors other than diet that influence CVD risk, such as exercise and genetics: “*…look I believe that there is some genetic factors involved, but I certainly believe that we can do a lot more with diet and exercise*” (female). Participants who rated diet as very important believed that diet is a significant moderator of other factors and is capable of overriding other significant risks in which personal actions shape health outcomes.

Very few participants believed that hereditary factors inevitably will prevail and counteract dietary and lifestyle intentions.

“*I think sometimes no matter what your diet is, if you’ve got a problem genetically or anything, it can more or less kind of help, but sometimes you just have that problem and that’s it, you know.*”

(female)

Several participants mentioned the importance of routine in adhering to a healthier diet. Planning meals and purchases, as well as preparing meals at home, were some of the elements of routine which were considered conducive to healthy eating.

“*Because I’m out so much every day and staying at different places, your diet just falls out of place...Routine [keeps me inline], because even waking up later and just having a late breakfast puts you out of place, but routine makes it easy and I always take fruit to school.*”

(female)

The most often-mentioned barrier to eating heart healthy foods was lack of convenience and the corresponding accessibility to healthy foods. Some mentioned lack of healthy options when eating out or while travelling.

“*Particularly if you go out and eat, you’re always getting lathered with chips. They’re just tasty, so you do tend to eat them...even if you get salad you’ll have steak and salad, you’ll still get chips. Yeah, so I probably tend to order steak and vegetables…Because that eliminates the chips.*”

(male)

Another participant felt they had limited access to good quality healthy foods that they would like to consume:

“*…look there’s not everything there that is what we would eat normally. So certainly isn’t your fish, the range of fish and things like that, that we like to eat… it’s a standalone supermarket. So if it’s not in the supermarket, it’s very inconvenient to go and get it elsewhere.*”

(male)

The concept of accessibility and convenience appeared to be a powerful motivator for consuming highly processed and unhealthier foods. Being time-poor was presented as a rationale for less healthy eating patterns, due to the extra time involved in preparing healthier meals: “*Sometimes it’s easier to grab something that’s not necessarily totally nutritious and often the raw natural foods require a bit more preparation than the instant stuff.*” (female) Portability and accessibility of “instant” lower quality foods was emphasized by a younger working woman, who identified it as an important obstacle to the consumption of healthier options.

Overall, there seemed to be an acceptance that life sometimes would “get in the way” of a healthy diet. Indeed, most participants appeared to engage in a cognitive balancing act, where the benefits of quick and easy food choices were used to justify less desirable dietary habits.

“*...everything I eat is not necessarily is what I would think is good for my heart. I mean, there’s got to be some things that are not good for it. But life goes on and often you’ve got to have something—well, I feel you’ve got to have some enjoyment out of life, and sometimes that goes to living on the edge, I suppose.*”

(male)

### 3.4. Family Member Influences on Adoption of Recommended Eating Patterns

Participants reported a range of circumstances where individual members exerted influence over family eating behaviours, either through a willingness to change, or through indicating resistance and providing challenges to achieving change. Participants described family members who were champions that supported at least some of the recommended eating patterns, as well as some who acted as saboteurs.

Several participants made reference to tastes and preferences being an important barrier to healthier eating patterns. These barriers varied between preferences for unhealthier alternatives established through lifelong habits and cultural/traditional culinary heritage, to reluctance of either self or partner to try new healthier foods. Indeed, for most participants where food preferences were a barrier, their male partner’s preference for other foods was the limiting factor.

Reluctance of a partner to change could have led to conflict but appeared to be resolved in one of two ways: either separate meals or conceding to the less healthy option. For one older woman, her husband’s reluctance to change dictated which meals were served:

“*…sometimes [wife’s name] makes something for herself that I don’t necessarily like, so she has another meal in the freezer for me or she will cook something different for me, you know?*”

(male)

Alternatively, she would prepare and eat less nutritious meals, as the additional effort and time involved in cooking separate meals acted as a powerful barrier to adoption of her own healthier eating patterns:

“*You tend to be a little bit lazy when you’re doing the cooking just for two… it just makes it easier to cook the same thing.*”

(female)

Some family members identified advantages in compromising their preferences. One man indicated:

“*…there might be some things that I don’t particularly like taste wise, but I know they are probably good for me. So I will eat them without too many qualms. If I complain too much, then it won’t happen and I will have to cook.*”

(male)

The advantage of following the lead of the ringleader in the presence of discrepancies between preferences and health behaviours was recognised by another husband:

“*[If she wasn’t as concerned as me, I would] probably become a little bit more complacent I would imagine. Having someone strong and up there to remind you is certainly beneficial... She’s the driving force.*”

(male)

Related to this dyadic effect within households was a prominent issue relating to “bad foods” being brought into the home environment, creating temptations for others. In most cases, one person in the household was clearly identified, by self and others, as the “perpetrator”:

“*I’m probably, maybe, a bigger offender than…I mean, I do mainly buy the food that comes into the house.*”

(female)

The majority of participants perceived their family as being able to openly discuss diet-related issues, however interviewees revealed that conflict was more likely avoided by circumventing confrontation:

“*I mean, after 30-odd years, you learn not to talk about things you don’t need to talk about, don’t you?*”

(male)

Another wife commented:

“*I probably don’t tell him [that he needs to lose weight]. He’s the one who says ‘oh, I need to lose a bit.’ I think when he had his knees hurt or his ankles hurt, I might have suggested that losing a bit of weight might help. He just says ‘I know’. So we don’t - I think we need to do things together to help, to motivate each other as far as that goes.*”

(female)

In the few instances in which participants alluded to the presence of food-related topics capable of eliciting conflict, there appeared to be an implicit understanding of what and how to bring up certain issues. Indeed, one participant used the idiom ‘walking on eggshells’ to describe the sensitivity of food related issues around his obese sister, iterating the fact that the sensitive nature of food related topics, for some, will prevent open discussion around health promoting issues.

Cooking different meals and trialling new recipes were overt strategies used to try and facilitate dietary changes. Some tried a more inclusive partnership approach to increasing support for making dietary changes:

“*He loves all the homemade stuff I make, all the soups that I make…he can’t cook, but he’s chopping up the vegetables for soup, so he can do that.*”

(female)

Others employed more covert strategies to facilitate adopting some of the dietary recommendations:

“*…he is very defensive about changes to the diet, and if it’s pointed out that something might be unhealthy he gets very defensive about it. I think he feels quite threatened that someone might change the status quo… because I work a lot, he does the shopping, so I have changed his shopping habits... But I have to be a bit clever about it… I just write him little lists and stuff like that and kind of plant the seed and I sort of display the fruits and things like that prominently around the place. So he knows that’s what’s getting eaten.*”

(female)

Several participants spoke of their boundaries of influence over the eating patterns of other family members. Beliefs relating to the extent of perceived influence guided their behaviours:

“*If he doesn’t like certain things, it’s a bit hard to make something different – two different types of meals…But sometimes I say “Oh no, you can try this”.*”

(female)

“*There’s times when I can influence that [the home environment] and times that I can’t. Where I could I would.*”

(male)

“*It goes in one ear and out the other when I say something. I think a lot of wives have the same problem.*”

(female)

Two participants recognised that their influence was not enough to achieve the desired behaviour change in their partners but identified a role for health professionals:

“*The funny thing with him is that he will really listen to a professional. He probably listens to me a little bit less …so he came home [after a dietetic consultation] and he’s actually been on a relatively low GI diet ever since…. he really respects people who know what they’re talking about.*”

(female)

“…*I think they need a professional other than me saying all the time, ‘You need to do this. You need to do that.’ He needs it coming from some other person who’s a professional*.”

(female)

## 4. Discussion

The current study intended to qualitatively explore risk perceptions and motivations to engage in dietary risk-reduction behaviours in families at increased risk of CVD to determine approaches that may be useful in engaging similar populations. The various influences of family members in adopting healthier eating patterns were also explored. Results indicate that risk perception alone may be inadequate to initiate and sustain dietary changes in this population. However, individual family members were shown to be both positive and negative influences on dietary patterns and may be utilised to enhance adherence to dietary recommendations.

Heart health concern was explored with both index and secondary recruits. This concern directly relates to threat appraisal, a major construct of PMT. There was a disparity between perceived and actual risk, with participants rarely estimating their true level of risk. Cardiovascular risk tends to be inaccurately or optimistically viewed and these results are similar to those with established CVD, familial hypercholesterolemia and asymptomatic populations, who have all been shown to have poor agreement between perceived and true CVD risk, with those at high risk particularly underestimating their actual risk [24,25,26]. This was most recently demonstrated in a multinational cohort of approximately 3500 participants from United States and Spain who experienced an acute myocardial infarction before 56 years of age. Only half (53%) of the patients considered themselves at risk of CVD, even though almost all (98%) had at least one risk factor [27]. In addition, those experiencing CVD events may understand their condition as acute, not chronic, with the effects limited in time [8]. According to PMT, for dietary changes to be made, an estimation of threat has to be perceived before coping options can be evaluated [28]. The under-estimation of risk at an individual level may be overcome through the use of the family “ringleader”. The results from this study showed that ringleaders saw the intervention as an opportunity to reduce risk not only for themselves, but actively recruited other members of their family to join them.

As an explanatory theory for behaviour relating to CVD health, the PMT provides that coping appraisal is used in conjunction with threat appraisal. Coping appraisal was explored through both response- and self-efficacy. Response efficacy was explored within the relationship of heart health and diet and self-efficacy was explored within barriers faced and the influence of family. Coping appraisal within the PMT includes the belief that taking the recommended steps will produce a positive response (response efficacy) [28]. In this study, many people who believed that “*you are what you eat*” described a relationship between diet and CVD within a more holistic understanding of lifestyle that included diet and exercise, but also apportioned a role for genetics.

It appears that many individuals believe they are already taking the necessary steps, such as taking medication and “eating well. Past surveys of the Healthy Eating Index, a guide to diet quality, have shown that approximately 40% of the surveyed population optimistically perceived their eating habits to be of better quality than they actually were, with only 40% evaluating their diet quality accurately [29]. A recent European study found that approximately one in five adults in their study sample overestimated their adherence to vegetable intake guidelines [30]. In addition, those who make small changes to their intake may consider it the appropriate level of change needed [9]. The results presented here showed interviewees to rationalise their need to take further steps to reduce their risk of heart disease based on their current symptoms or lack thereof.

Factors such as social supports and unhelpful social contacts can affect the motivations of those with CVD and contribute to poor adherence to dietary recommendations [9,10,11]. Although an individual’s intention to perform an action may have been verbalised, whether the action was performed was likely to have been dependent upon other factors. The barriers to healthy eating encompassed a range of issues: the complex relationships with partners, the need to deal with partner’s food preferences, individualised eating, with occasions of eating away from home being common. Family members were either a significant form of support or barrier to improving dietary patterns. These findings are similar to others: Aggarwal et al. showed that in family members of people with established CVD, those with low social support are less likely to adhere to a therapeutic diet [31]. This may also be related to socio-economic status. Some of the interviewees were from households with substantially lower incomes than the median income of the region from which intervention participants were recruited. Lawrence et al. reported that women from lower socio-economic backgrounds exhibited less control over healthy eating patterns of other family members, such as children or spouse, compared to women with more advantaged backgrounds [32]. Less control over eating patterns was shown to occur in some of these families where a woman would prepare multiple meals or compromise her own decision to eat healthy foods. Findings from health habits of married individuals demonstrate that, when one spouse initiates a new health behaviour, it significantly increases the odds of the other in adopting the same behaviour, particularly for behaviours such as decreasing smoking (odds ratio (OR): 5.65 men, 5.21 women) and drinking (OR: 5.64 men, 5.58 women), and that readiness to change behaviour is similar between married couples [12,14]. Evans showed that family is a bigger influence on changes in dietary behaviours than friends for African American women, whether offering encouragement or criticisms [33]. Social support can be extended to social contacts outside of the family unit, with these also creating a supportive or negative influence [10].

Eating away from the home, lack of time and individualised eating were some of the barriers mentioned in the results. These are aligned with food choice coping strategies and affect dietary quality and nutritional adequacy [34]. Blake et al. showed that these coping strategies are related to work, marital status and the number of children. Those with a lack of time may miss meals or choose foods requiring less preparation, leading to reduced intakes of quality grains and milk [34]. Those preparing individualised meals tended to eat less dark green and orange vegetables [34]. This is of concern in regards to populations looking to improve heart health, as unrefined and highly coloured plant and vegetable matter are associated with eating patterns such as the Mediterranean diet, which have been demonstrated to improve CVD outcomes [4,35].

Strengths and limitations of this study include the qualitative design, which allowed participants to express in their own words their experiences of undertaking measures to prevent CVD by dietary means. The interviewer being known to participants is both a strength and a weakness, having developed contact during the study intervention period. Prior contact allowed rapport to be previously established between interviewer and interviewee, thereby allowing the interviews to be performed by telephone, although it may also have biased the opinions that were expressed. Non-verbal cues that may have been incorporated in a face-to-face interview were also not possible. However, choice of time and location for the interview to take place allowed for the participant to choose a comfortable situation to increase their own privacy. The sample size interviewed was small and limited to a regional geographic area. Therefore, these findings are not generalizable or representative of other populations. In addition, participants previously participated in a CVD risk reduction intervention and should be expected to have an increased knowledge of the role of diet in CVD risk management.

## 5. Conclusions 

Although the PMT is a suitable theory for this population at elevated risk for CVD due to family histories of CVD and shared environments, the results demonstrate the importance of understanding risk perception, as variation was seen between perceived and actual CVD risk. Family members may act as enablers or barriers to adherence dependent upon their understanding of risk and how they perceive the “healthiness” of their personal and/or shared current diet. Participants described a strong belief in the concept of moderation, but expressed persistent barriers to improving their dietary intakes. Inclusion of and accountability to family members was reported as a strong motivator.

This study has implications for practitioners aiming to improve adherence to dietary recommendations in those at increased risk of CVD and their families. Firstly, self-perception of risk may not be associated with objective risk and may be insufficient to engage families in dietary changes. Other more powerful motivators may be required. The issues of accessibility to health foods, routine and being “time-poor” may be addressed concurrently by stressing the importance of planning ahead and developing new routines where necessary. It may also be constructive to include those who act as barriers to improvements or the “perpetrators” of unhealthier eating habits in the counselling sessions. Benefits may also be achieved by enlisting the support of individual family members, particularly the family “ringleader”, to adopt the recommended dietary patterns and capitalise on intra-family accountability. Further to this, there is a need for future research to investigate methods of identifying influential family member/s, the extent of influence and how it affects dietary choices.

## Figures and Tables

**Table 1:** Salient points that emerged from the areas of enquiry.

Areas of Enquiry	Salient Points
Motivations	Importance of family unit
	Ringleaders and secondary recruits
Risk perceptions	CVD risk perception in index participants
	CVD risk perception in secondary recruits
Heart health and diet	Relative importance of diet
	Routine
	Accessibility to healthy foods
Influence of family on eating patterns	Working within confines of family roles and preferences
	Inferred, not discussed
	Overt and covert approaches
	Recognised boundaries of influence

CVD: Cardic Vascular Disease

## Supplementary Materials

The following are available online at www.mdpi.com/2227-9032/4/4/74/s1, Table S1: Interview script.
