# Supplementary material for: The Role of Family in a Dietary Risk Reduction Intervention for Cardiovascular Disease
Source: Healthcare (Basel). 2016 Sep 30;4(4):74. doi: 10.3390/healthcare4040074 (PMC5198116; doi:10.3390/healthcare4040074)
Supplement: Supplementary file 1 [file healthcare-04-00074-s001.pdf]

**Supplementary Table 1: Interview Script**
**Topic: Why did you volunteer?**
**1a)** Let's start by choosing a name other than your real name. We will use this in the interview instead of your real name to protect your privacy. Any name you want to use is ok. It could be your favourite actor, your favourite cartoon character, etc.<br><br>**1b)** What name would you like to be called? Could you tell me something about your reasons for choosing that name? [keep brief]<br><br>**2)** Ok. [insert name]. Let's start by talking about how you heard about the study?<br><br>**3a)** What were your reasons for signing up?<br>***Probe:***<br>- Was there one family member that wanted to do this more than others? *If yes, who?*<br>**4)** What made you want to do this with your family?<br>***Prompts that can be used:***<br>*- social*<br>*- health*<br>*- support or concern for other family members*<br>*- encouraged by someone else in the family*<br><br>**5)** How would you rate your concern about your heart health, if 1 was not concerned and 5 was very concerned?<br>***Probe:***<br>*-Why have you chosen **X**?*<br><br>**6)** What impact, if any, do you think eating habits have on your heart health, if 1 was what I eat makes no difference to my heart health, and 5 was everything I eat makes a difference to my heart health?<br>***Probe:***<br>*-Why have you chosen **X**?*
**Topic: Eating well – is it a problem?**
7. a) How would you define "healthy food"?<br>b) What makes food healthy or unhealthy?<br>c) Can you give me examples of healthy foods or unhealthy foods?<br>**8.** What barriers, if any, make it hard for your family to eat heart healthy foods?<br>***Probe:***<br>- Are any unhealthy foods purchased and bought into the house that think shouldn't be there? *(give examples)*<br>- Do you think this is a problem? Why?<br>**9.** How would you rate the healthiness of your food patterns before you started the study, based on a scale of 1 to 10 with 10 being the healthiest?<br>**10.** And how would you rate it now?<br>***Probe:***<br>- Please tell me the reasons your rating has changed / stayed the same?<br>- **If rating has increased:** What have you done to make your diet healthier?<br>- Has the study changed the way you think about what a healthy diet is? *(give examples)*

Topic: Family dynamics

11. On a scale of 1 to 5, with one being not concerned and 5 being very concerned, how concerned are others in your immediate family about what they eat?

12. On the same rating scale, how concerned are others in your family about their heart health?

Probe:

- Why have you given them these ratings?

If different level of concern as given in Question 5:

13) What do you do when your significant others are not as concerned about their heart health or food intake as you are?

If same level of concern as given in Question 5:

13) What would you do if any of your significant others were not as concerned as you are their heart health?

Prompts that can be used:

- E.g. address the issue with the person by talking about it or change the home environment

14. Are there eating topics that are avoided in your household because they might cause conflict? ***Yes / No***

Prompts that can be used:

- E.g. the types or amounts of certain foods, or foods that someone thinks might be damaging to a another person's health

Prompts that can be used if YES:

- Can you tell me what the topics are?
- What usually happens when these topics are bought up?

15. When someone in the family has a health problem, what does your family usually do?

Prompts that can be used:

- Is it discussed, avoided, plan changes…
- Is there someone in the family that other family members go to, to talk about their health issues? (why?)
- What do **YOU** do if someone in the family has a health problem?

Topic: Role of the health professional in topics that may be sensitive in nature

16. If there was a health problem related to food in your family that could cause potential conflict, is there one kind of health professional that you would choose over another to help with this?

Prompts that can be used:

- Do you think it makes a difference which health professional addresses the issue? For example, GP, dietitian, nurse or pharmacist?
- Is there a health professional that you would trust more than another in this situation?

Probe:

- Why do you think *(X health professional)* will be the best person for this sensitive topic?
- How do you think that talking this over with *(X health professional)* will change the way this problem is dealt with within your family?

Topic: Summary

So, to summarise all that we talked about tonight, you've said **[summarise briefly]**. Is this accurate or is there something that you would like to change or clarify? Have we missed anything that you would like to talk about and haven't had a chance to say?

Thank you for your time tonight, it is very much appreciated. You will receive your gift voucher in the mail sometime in the next few days. Have a good evening.
